# The Notions of Fuzzy Set on Pseudo Quasi Ordered Residuated Systems

**DOI:** 10.12688/f1000research.165259.2

**Published:** 2025-07-24

**Authors:** Yeshiwas Mebrat Gubena, Teferi Getachew Alemayehu

**Affiliations:** 1 Mathematics, Bahir Dar University Department of Mathematics, Bahir Dar, Amhara, Ethiopia; 2 Mathematics, Debre Berhan University, Debre Birhan, Amhara, Ethiopia

**Keywords:** associative fuzzy filter; pseudo quasi-order residuated system; Comparative fuzzy filter; fantastic fuzzy filter; Quasi- order residuated system.

## Abstract

This paper introduces the concept of fuzzy filters and 2-fuzzy filters of a quasi-ordered residuated system K . This research explores comparative, normal, implicative and associative fuzzy filters within a quasi-ordered residuated system. It establishes the sufficient conditions under which a comparative fuzzy filter in K qualifies as a normal fuzzy filter and vice-versa. Furthermore, the study demonstrates that the collection of all comparative fuzzy filters in K constitutes a complete lattice. The notion of fuzzy filters of a pseudo quasi-ordered residuated system is introduced and the analysis extends to fantastic, comparative, associative and implicative fuzzy filters within a pseudo quasi-ordered residuated framework. Finally, it is proven that an associative fuzzy filter in K inherently satisfies the criteria for both implicative and comparative fuzzy filters.

## 1. Introduction

Quasi-ordered residuated system, conceptualized by S. Bonzio and I. Chajda, ^1^ is defined as commutative residuated integral monoid structured under a quasi-ordered. Recent advancements have significantly expanded the theoretical framework of quasi-order (pseudo quasi-ordered) residuated system, particularly concerning ideals and filters. ^2– 4^ Daniel A. Romano has contributed substantially to this domain by investigating the substructures of filters within these algebraic systems. ^2^ His subsequent work led to the introduction and detailed examination of various classes of filters, including implicative filters, ^5^ associated filters, ^4^^,^^6^ and comparative filters. ^7^

Furthermore, a formal connection between comparative filters and implicative filters within this algebraic framework has been elucidated. The foundational framework of fuzzy set theory, conceptualized as a generalization of classical set theory, was pioneered by Zadeh. ^8^ In the realm of algebraic structures, Rosenfield ^9^ initiated seminal research on the fuzzification of algebraic objects, particularly in the context of fuzzy groups. Building upon this groundwork, Y. Bo et al. ^10^ and Swamy et al. ^11^ systematically established the theoretical foundation for fuzzy ideals of lattices. Further advancing this field, C. Santhi Sundar Raj et al. ^12^ introduced the notion of fuzzy prime ideals in almost distributive lattices (ADLs), broadening the scope of fuzzy algebraic studies.

In 1998, U. M. Swamy and D. Viswanadha Raju ^11^ expanded the theoretical landscape by formalizing fuzzy ideals and fuzzy congruences in distributive lattices, demonstrating a one-to-one correspondence between the lattices of fuzzy ideals and that of fuzzy congruences. Subsequent investigations by U. M. Swamy et al. ^13^ explored the concept of L-fuzzy filters within the framework of ADLs, further enriching the algebraic discourse on fuzzy theory.

## 2. Preliminaries

This section introduces essential definitions and results that will be utilized throughout the paper. The abbreviations QORS and PsQORS stands for quasi-ordered residuated systems and pseudo quasi-ordered residuated systems respectively, unless otherwise specified.

Definition 2.1. ^1^ A residuated relational system is defined as a structure K=(K,·,→,1,R) , where (K,·,→,1) is an algebra of type (2 *,* 2 *,* 0) and R is a binary relation on *K* satisfying the following properties:

(1)
(K,·,→) is a commutative monoid,
(2)
(a,1)∈R , for all a∈K ,
(3)
(a·b,c)∈R⇔(a,b→c)∈R , for all a,b,c∈K .

We refer the operations ‘ · ’ as multiplication and ‘ → ’ as its residuum.

Definition 2.2. ^1^ A QORS is a residuated relational system K=(K,·,→,1,≼) , where ≼ is a quasi-ordered relation defined on the monoid (K,·) .

Proposition 2.3. ^1^ *Let* K *be a QORS. Then, for any* a,b,c∈K *,*

(1)
a≼b⇒a·c≼b·c *and* c·a≼c·b *,*
(2)
a≼b⇒b→c≼a→c *and* c→a≼c→b *,*
(3)
a·b≼a *and* a·b≼b *,*
(4) *a →* ( *b → c*) ≼ *b →* ( *a → c*) *,
*
(5) *a → b* ≼ ( *b → c*) *→* ( *a → c*) *.
*

Definition 2.4. ^2^ For a non-empty subset J of a QORS, K we say that it is a filter in K if it satisfies the following conditions:

(1)
a∈J and a≼b⇒b∈J ,
(2)
a∈J and *a → b* ⇒ b∈J ,

for all a,b∈K .

Definition 2.5. ^5^ A non-empty subset J of a QORS, K is implicative filter in *K* if the following conditions hold for any a,b,c∈K :

(1)
a∈J and a≼b⇒b∈J ,
(2)
a→(b→c)∈J and a→b∈F⇒a→c∈J .

Theorem 2.6. ^7^ *Let J be a non-empty subset of a QORS*, K *such that* a∈J *and* a≼b *implies* b∈J *for all* a,b∈K *. If* (a→(b→(b→c)))∈J *, then* b∈J *.*

Definition 2.7. ^7^ For a non-empty subset J of a QORS, K we say that it is comparative filter in K if the following conditions hold for any a,b,c∈K :

(1)
a∈J and a≼b⇒b∈J ,
(2)
a→((b→c)→b)∈J and ∈J⇒b∈J .

Theorem 2.8. ^7^ *A comparative filter of a QORS*, K *is a filter of* K *.*

Definition 2.9. ^4^ A pseudo quasi-ordered relational system is a structure PK=(K,·,→,⇝,1,≼) where an algebra (K,·,→,⇝,1) is an algebra of type (2,2,2,0) and ≼ is a quasi-ordered in *K* satisfying the following properties:

(1)
(K,·,1) is a monoid,
(2)
a≼1 , for all a∈K ,
(3)
a·b≼c⇔a≼b→c for all a,b,c∈K ,
(4)
a·b≼c⇔b≼a⇝c for all a,b,c∈K .

Recall that, for any set *K* a function ϵ : K→[0,1] is called a fuzzy subset of *K*, where [0 *,* 1] is a unit interval. For every a∈[0,1] , the level subset ϵ of *K* is defined by $\epsilon_{a}$ ={b∈K:a≤ϵ(b)} .

Definition 2.10. ^10^ A fuzzy subset ϵ of a bounded lattice K is said to be a fuzzy ideal of *K*, if for all a,b∈K

(1)
ϵ (0) = 1,
(2)
ϵ(a∧b)=ϵ(a)∨ϵ(b) ,
(3)
ϵ(a∨b)=ϵ(a)∧ϵ(b) .

Definition 2.11. ^13^ A fuzzy subset ϵ of a bounded lattice *K* is said to be a fuzzy filter of *K*, if for all a,b∈K

(1)
ϵ(1)=1 ,
(2)
ϵ(a∧b)=ϵ(a)∧ϵ(b) ,
(3)
ϵ(a∨b)=ϵ(a)∨ϵ(b) .

## 3. Fuzzy Filters of Quasi-Ordered residuated systems

In the rest part of the manuscript, for any fuzzy subset ϵ of K and for all a,b∈K the condition a≼b⇒ϵ(a)≤ϵ(b) is denoted by (QF).

Theorem 3.1. *Let* K=(K,.,→,1,≼) *be a QORS and* ϵ *be a fuzzy subset on* K *satisfying (QF). Then, for any* a,b∈K *,* ϵ(a)∧ϵ(b)≥ϵ(a·b) *.*

Proof.
Suppose K be a QORS and ϵ be a fuzzy subset of K satisfying (QF). Clearly, a·b≼a and a·b≼b for any a,b∈K . Thus, from the assumption it follows that ϵ(a)≥ϵ(a·b) and ϵ(b)≥ϵ(a·b) so that ϵ(a)∧ϵ(b)≥ϵ(a·b) .      □

Theorem 3.2. *Let* K *be a QORS and* ϵ *be a fuzzy subset of* K *satisfying (QF). Then,* ϵ(a→b)≥ϵ(a) *for all* a,b∈K *.*

Proof.
Let *ϵ* be a fuzzy subset of K and a,b∈K such that a≼b . Then, as a·a≼a *,* we have a·a≼b and hence a≼a→b . Therefore, ϵ(a→b)≥ϵ(a) .      □

Theorem 3.3. *Let ϵ be a fuzzy subset of a QORS*, K *satisfying (QF) and* a≼b→c *for all* a,b,c∈K *. Then,* ϵ(c)≥ϵ(a·b) *iff* ϵ(b)≥ϵ(a) *.*

Proof.
Let *ϵ* be a fuzzy subset on K and a,b,c∈K such that a≼b and a≼b→c . So, a·b≼c and hence ϵ(c)≥ϵ(a·b) . Conversely, let a≼b and a≼b→c implies that ϵ(c)≥ϵ(a·b) . Obviously, a·1≼b so that a≼1→b . Consequently, ϵ(b)≥ϵ(a·1)=ϵ(a) .      □

Remark 3.4. *For a fuzzy subset ϵ of a QORS*, K *,* ϵ(1)≥ϵ(a) *for all* a∈K *.*

Definition 3.5. A fuzzy subset *ϵ* of a QORS, K satisfying (QF) is said to be a fuzzy filter of K whenever ϵ(1)=1 and ϵ(b)≥ϵ(a)∧ϵ(a→b) , for every a,b∈K .

Example 3.6. *Let* K={1,2,3,4} *and the operations ‘·’ and ‘→’ be defined on* K *as follows:*

**Table:** 

*.*	*1*	*2*	*3*	*4*
*1*	*1*	*2*	*3*	*4*
*2*	*2*	*2*	*2*	*4*
*3*	*3*	*2*	*2*	*4*
*4*	*4*	*4*	*4*	*4*

**Table:** 

*→*	*1*	*2*	*3*	*4*
*1*	*1*	*2*	*3*	*4*
*2*	*1*	*1*	*1*	*1*
*3*	*1*	*2*	*1*	*4*
*4*	*1*	*2*	*3*	*1*

*Then,* K *= (K, . , →, 1, ≼) is a QORS where ‘* ≼ *’ is defined by:*

≼≔{(1,1),(2,2),(3,3),(4,4),(4,1),(3,1),(2,1),(2,3),(2,4)}.

*Now, define a fuzzy subset* ϵ *of* K *by:*

ϵ(1)=1,ϵ(2)=0.5,ϵ(3)=0.6,ϵ(4)=0.8.

*Hence, ϵ is a fuzzy filter of* K *.*

Theorem 3.7. *The family* F(K) *of all fuzzy filters in a QORS*, K *forms a complete lattice.*

Proof.
Let ${\left\{\epsilon_{i}\right\}}_{i\in △}$ be a family of all fuzzy filters in a QORS, K . Clearly, ${\left\{\epsilon_{i}\right\}}_{i\in △}$ is a partially ordered set under set inclusion where △ is an indexed set. Let a,b∈K such that a≼b . Since $\epsilon_{i}$ is a fuzzy filter of K , $\epsilon_{i}\left(b\right)\geq \epsilon_{i}\left(a\right)$ for all i∈△ . Thus, ${⋂}_{i\in △}\epsilon_{i}\left(b\right)\geq {⋂}_{i\in △}\epsilon_{i}\left(a\right)$ so that condition 1 of Definition 3.5 is satisfied.

Clearly, for any a,b∈K ,

$${⋂}_{i\in △}\epsilon_{i}\left(a\right)\leq \epsilon_{i}\left(a\right)\;\text{and}\;{⋂}_{i\in △}\epsilon_{i}\left(a\to b\right)\leq \epsilon_{i}\left(a\to b\right).$$

From the fact that $\epsilon_{i}$ is a fuzzy filter for each i∈△ , we have

$${⋂}_{i\in △}\epsilon_{i}\left(a\right)\wedge {⋂}_{i\in △}\epsilon_{i}\left(a\to b\right)\leq \epsilon_{i}\left(a\right)\wedge \epsilon_{i}\left(a\to b\right)\leq \epsilon_{i}\left(b\right),$$

so that

$${⋂}_{i\in △}\epsilon_{i}\left(a\right)\wedge {⋂}_{i\in △}\epsilon_{i}\left(a\to b\right)\leq {⋂}_{i\in △}\epsilon_{i}\left(b\right).$$

This shows that condition 2 of Definition 3.5 is satisfied. Hence, ${⋂}_{i\in △}\epsilon_{i}$ is a fuzzy filter of K . Now, consider fuzzy filters of K which contains ${⋃}_{i\in △}\epsilon_{i}$ and denote the family of such filters by G .

Clearly, ⋂G is the smallest fuzzy filter which contains ${⋃}_{i\in △}\epsilon_{i}$ . Taking

$${⨆}_{i\in △}\epsilon_{i}=⋂G\;\text{and}\;{⨅}_{i\in △}\epsilon_{i}={⋂}_{i\in △}\epsilon_{i}$$

makes the algebra ( K *,* ⨅ *,* ⨆ ), a complete lattice.      □

Corollary 3.8. *Let* K *be a QORS and* ϵ *be a fuzzy subset of* K *. Then, there exists a minimal fuzzy filter of* K *which contains* ϵ *.*

Definition 3.9. A fuzzy subset ϵ of K satisfying (QF) is said to be a 2-fuzzy filter in K if ϵ(b·c)≥ϵ(a→c)∧ϵ((a→b)→c) for all a,b,c∈K .

Theorem 3.10. *Let* K *be a QORS. Then, every 2-fuzzy filter of* K *is a fuzzy filter of* K *.*

Proof.
Let ϵ be a 2-fuzzy filter of a QORS, K . For any a,b∈K , we show that ϵ(b)≥ϵ(a)∧ϵ(a→b) . From Theorem 3.2, ϵ(a→b)≥ϵ(a) . So, as a→b≼1 and ϵ(1)≥ϵ(a→b) , then ϵ((a→b)→1)≥ϵ(a→b) . Also, as a≼1 and ϵ(1)≥ϵ(a), then ϵ(a→1)≥ϵ(a) . Thus,

ϵ(a→1)∧ϵ((a→b)→1)≥ϵ(a)∧ϵ(a→b).

By the condition of 2-fuzzy filters, we get

ϵ(b·1)≥ϵ(a→1)∧ϵ((a→b)→1)≥ϵ(a)∧ϵ(a→b),

which shows that

ϵ(b)≥ϵ(a)∧ϵ(a→b).

Therefore, ϵ is a fuzzy filter of K .      □

## 4. Implicative, comparative and normal fuzzy filters of QORS and PsQORS

This segment explores various fuzzy filter structures, including implicative, comparative, normal, fantastic, and associative fuzzy filters, within the contexts of QORS and PsQORS.

Definition 4.1. A fuzzy subset ϵ of a QORS, K satisfying (QF) is said to be an implicative fuzzy filter of K if ϵ(a→c)≥ϵ(a→(b→c))∧ϵ(a→b) for all a,b,c∈K .

Example 4.2. *Let* K={a,b,c,1} *and the operations ‘·’ and ‘→’ be defined on K as follows:*

**Table:** 

*·*	*a*	*b*	*c*	*1*
*a*	*a*	*a*	*c*	*a*
*b*	*a*	*a*	*c*	*b*
*c*	*c*	*c*	*c*	*c*
*1*	*a*	*b*	*c*	*1*

**Table:** 

*→*	*a*	*b*	*c*	*1*
*a*	*1*	*1*	*1*	*1*
*b*	*a*	*1*	*c*	*1*
*c*	*a*	*b*	*1*	*1*
*1*	*a*	*b*	*c*	*1*

*Then,* K =(K,.,→,1,≼) *is a QORS where ‘* ≼ *’ is defined by:*

≼≔{(1,1),(a,a),(b,b),(c,c),(c,1),(b,1),(a,1),(a,b),(a,c)}.

*Now, define a fuzzy subset* ϵ *by:*

ϵ(1)=1=1(b),ϵ(a)=0.7andϵ(c)=0.8.

*It is simple to show that* ϵ *is an implicative fuzzy filter of* K *.*

Theorem 4.3. *Let* ϵ *be an implicative fuzzy filter of a QORS*, K *. Then, for any* a,b∈K,ϵ(a→b)≥ϵ(a→(a→b)) *.*

Proof.
Let ϵ be an implicative fuzzy filter of a QORS, K . Let a,b∈K , then taking b=a in the hypothesis of Definition 4.1, we get

ϵ(a→c)≥ϵ(a→(a→c))∧ϵ(a→a).

Since 1·a≼a so that 1≼(a→a) and we have ϵ(a→a)≥ϵ(1) . Thus,

$$\begin{array}{c} \epsilon \left(a\to c\right)\;\geq \epsilon \left(a\to \left(a\to c\right)\right)\wedge \epsilon \left(1\right)\\ \geq \epsilon \left(\left(a\to \left(a\to c\right)\right)\cdot 1\right)\\ =\epsilon \left(a\to \left(a\to c\right)\right) \end{array}$$

This proves the theorem.      □

Lemma 4.4. *Let* ϵ *be a fuzzy subset on a QORS*, K *satisfying (QF). Then,* ϵ(1→a)=ϵ(a) *for all* a∈K *.*

Proof.
Let ϵ be a fuzzy subset of a QORS, K and a∈K . We have a≼1→a and 1→a≼a . Thus by hypothesis ϵ(a)≤ϵ(1→a) and ϵ(1→a)≤ϵ(a) so that ϵ(a)=ϵ(1→a) .      □

Theorem 4.5. *Every implicative fuzzy filter of a QORS*, K *is a fuzzy filter of* K *.*

Proof.
Let *ϵ* be an implicative fuzzy filter of a QORS, K and a,b∈K such that a≼b. From Lemma 4.4, we have ϵ(b)=ϵ(1→b) and from the hypothesis of Definition 4.1, we get

ϵ(b)=ϵ(1→b)≥ϵ(1→(a→b))∧ϵ(1→a).

Hence,

ϵ(b)≥ϵ(a→b)∧ϵ(a).

Therefore, ϵ is a fuzzy filter of K .      □

Lemma 4.6. *Let* ϵ *be a fuzzy subset of a QORS*, K *and* a,b,c∈K *. Then, (QF) holds iff for* a≼b→c,ϵ(c)≥ϵ(a·b) *.*

Proof.
The forward proof is trivial. Conversely, let a,b,c∈K such that if a≼b→c , then ϵ(c)≥ϵ(a·b) . Let a,b∈K such that a≼b. This is the same as a·1≼b . Thus, a≼1→b . Then, by the hypothesis we obtain that ϵ(b)≥ϵ(a·1)=ϵ(a) .      □

Lemma 4.7. *Let* ϵ *be a fuzzy subset of a QORS*, K *satisfying (QF). Then, for any* a,b,c∈K,ϵ(a→(b→c))=ϵ(b→(a→c)) *.*

Proof.
Let ϵ be a fuzzy subset of a QORS, K satisfying (QF). Then, for any a,b,c∈K ,

a→(b→c)≼a·b→c=b·a→c≼b→(a→c).

Thus,

ϵ(a→(b→c))≤ϵ(b→(a→c)).

Similarly,

ϵ(b→(a→c))≤ϵ(a→(b→c)).

Therefore,

ϵ(a→(b→c))=ϵ(b→(a→c)).

      □

Theorem 4.8. *Let* ϵ *be a fuzzy subset of a QORS*, K *satisfying (QF). If* ϵ(b→c)≥ϵ(a)∧ϵ(a→(b→(b→c))) *for any* a,b,c∈K *, then ϵ is an implicative fuzzy filter of* K *.*

Theorem 4.9. *Let* ϵ *be a fuzzy subset of a QORS*, K *satisfying (QF) and let* a,b∈K *such that*

(1)
ϵ(b)≥ϵ(a)∧ϵ(a→b)

(2)
ϵ(a→b)≥ϵ(a→(a→b)) *.*

*Then,* ϵ *is an implicative fuzzy filter of* K *.*

Proof.
Let ϵ be a fuzzy subset of a QORS, K satisfying the given conditions and let a,b,c∈K . Using (2), we have

ϵ(b→c)≥ϵ(b→(b→c)).

Also, by (1) we have

ϵ(b→(b→c))≥ϵ(a)∧ϵ(a→(b→(b→c))).

Thus,

ϵ(b→c)≥ϵ(a)∧ϵ(a→(b→(b→c))).

Therefore, by Theorem 4.8, ϵ is an implicative fuzzy filter of K .      □

Theorem 4.10. *A fuzzy subset* ϵ *of a QORS*, K *is an implicative fuzzy filter iff* $\epsilon_{\alpha }$ *is an implicative filter of* K *, for all* α∈[0,1].

Proof.
Suppose ϵ be an implicative fuzzy filter of a QORS, K . Let $a\to \left(b\to c\right)\in \epsilon_{\alpha }$ and $\to b\in \epsilon_{\alpha }$ , for any a,b,c∈K and for all α∈[0,1] . So, ϵ(a→(b→c))≥α and ϵ(a→b)≥α . Thus,

ϵ(a→c)≥ϵ(a→(b→c))∧ϵ(a→b)≥α∧α=α

in order that $a\to c\in \epsilon_{\alpha }$ . Hence, $\epsilon_{\alpha }$ is an implicative filter of K . Now, let $\epsilon_{\alpha }$ be an implicative filter of K and let

ϵ(a→(b→c))∧ϵ(a→b)=α.

Thus, ϵ(a→(b→c))≥α and ϵ(a→b)≥α so that

ϵ(a→c)≥α=ϵ(a→(b→c))∧ϵ(a→b).

Therefore, ϵ is an implicative fuzzy filter of K .      □

Definition 4.11. A fuzzy subset ϵ of a QORS, K satisfying (QF) is said to be a comparative fuzzy filter of K if ϵ(b)≥ϵ(a)∧ϵ(a→((b→c)→b)) for all a,b,c∈K .

Example 4.12. *Let* K={a,b,c,d,1} *and the operations ‘ ^.^’ and ‘→’ be defined on* K *as follows:*

**Table:** 

*·*	*a*	*b*	*c*	*d*	*1*
*a*	*a*	*d*	*c*	*d*	*a*
*b*	*d*	*b*	*d*	*d*	*b*
*c*	*c*	*d*	*c*	*d*	*c*
*d*	*d*	*d*	*d*	*d*	*d*
*1*	*a*	*b*	*c*	*d*	*1*

**Table:** 

*→*	*a*	*b*	*c*	*d*	*1*
*a*	*1*	*b*	*c*	*d*	*1*
*b*	*a*	*a*	*c*	*c*	*1*
*c*	*1*	*b*	*1*	*b*	*1*
*d*	*1*	*1*	*1*	*1*	*1*
*1*	*a*	*b*	*c*	*d*	*1*

*Then,* K=(K,.,→,1,≼) *is a QORS where ‘* ≼ *’ is defined by:*

≼≔{(a,a),(a,1),(b,b),(b,1),(c,a),(c,1),(d,a),(d,b),(d,c),(d,1),(1,1)}.

*Now, define a fuzzy subset* ϵ *of* K *by:*

ϵ(a)=1=ϵ(1),ϵ(b)=0.7,ϵ(c)=0.9andϵ(d)=0.6.

*Thus,* ϵ *is a comparative fuzzy filter of* K *.*

Theorem 4.13. *Let* ϵ *be a comparative fuzzy filter of a QORS*, K *. Then, for any* b,c∈K,ϵ(b)≥ϵ((b→c)→b).

Proof.
Let ϵ be a comparative fuzzy filter of a QORS, K and b,c∈K . Then, from the assumption of Definition 4.11

ϵ(b)≥ϵ(1)∧ϵ(1→(b→(c→b))).

From Lemma 4.4, we obtain that

ϵ(b)≥ϵ(1)∧ϵ((b→c)→b)=ϵ((b→c)→b).

     □

Remark 4.14. *If* ϵ *is a comparative fuzzy filter of a QORS*, K *and* a,b,c∈K *, then* ϵ(b)≥ϵ(a)∧ϵ((b→c)→(a→b)).

Theorem 4.15. *A comparative fuzzy filter* ϵ *of a QORS*, K *is a fuzzy filter of* K *.*

Proof.
Let ϵ be a comparative fuzzy filter of a QORS, K and a,b,c∈K . Then,

$$\begin{array}{c} \epsilon \left(b\right)\geq \epsilon \left(a\right)\wedge \epsilon \left(a\to \left(\left(b\to c\right)\to b\right)\right)\\ \;=\epsilon \left(a\right)\wedge \epsilon \left(\left(b\to c\right)\to \left(a\to b\right)\right). \end{array}$$

Since a→b≼(b→c)→(a→c) , we get

ϵ((b→c)→(a→c))≥ϵ(a→b).

Thus,

ϵ(b)≥ϵ(a)∧ϵ((b→c)→(a→b))≥ϵ(a)∧ϵ(a→b),

which shows that ϵ is a fuzzy filter of K .      □

Theorem 4.16. *Let* ϵ *be a fuzzy filter of* K *. Then* ϵ *is a comparative fuzzy filter of* K *iff* ϵ(b)≥ϵ((b→c)→b) *for all* a,b,c∈K *.*

Proof.
The proof of the forward direction follows from Theorem 4.13. Now, let ϵ be a fuzzy filter of K , and ϵ(b)≥ϵ((b→c)→b) for all b,c∈K . By the given condition of Definition 3.5, we have ϵ((b→c)→b)≥ϵ(a)∧ϵ(a→((b→c)→b)) for all a,b,c∈K . Thus,

ϵ(b)≥ϵ((b→c)→b)≥ϵ(a)∧ϵ(a→((b→c)→b)).

So, ϵ is a comparative fuzzy filter of K .      □

Theorem 4.17. *Let* K *be a QORS such that* a→b=((a→b)→b)→b *for all* a,b∈K *. If* ϵ *is a comparative fuzzy filter of* K *, then* ϵ((b→a)→a)≥ϵ((a→b)→b) *, for all* a,b∈K *.*

Proof.
Suppose ϵ is a comparative fuzzy filter of K and a,b∈K . Then, from Theorem 4.16, we obtain that

$$\begin{array}{c} \epsilon \left(\left(b\to a\right)\to a\right)\;\geq \epsilon \left(\left(\left(\left(b\to a\right)\to a\right)\to b\right)\to \left(\left(b\to a\right)\to a\right)\right)\\ \;=\epsilon \left(\left(\left(\left(a\to b\right)\to b\right)\to b\right)\to \left(\left(b\to a\right)\to a\right)\right)\;\\ \;=\epsilon \left(\left(a\to b\right)\to \left(\left(b\to a\right)\to a\right)\right)\\ \;=\epsilon \left(\left(b\to a\right)\to \left(\left(a\to b\right)\to a\right)\right). \end{array}$$

Since

(a→b)→b≼(b→a)→((a→b)→a)),

we obtain that

ϵ((b→a)→((a→b)→a)))≥ϵ((a→b)→b)

so that ϵ((b→a)→a)≥ϵ((a→b)→b). □

Theorem 4.18. *Let* ϵ *be an implicative fuzzy filter of a QORS*, K *such that* ϵ((b→a)→a)≥ϵ((a→b)→b) *for all* a,b∈K *. Then,* ϵ *is a comparative fuzzy filter of* K *.*

Proof.
Let ϵ be an implicative fuzzy filter of K and a,b∈K such that

ϵ((b→a)→a)≥ϵ((a→b)→b).

Clearly, ϵ is a fuzzy filter of K , i.e., ϵ(b)≥ϵ(a)∧ϵ(a→b) . Proving ϵ(b)≥ϵ(b→a)→b, completes the proof of the theorem. Now, consider the fact that, a→b≼(b→c)→(a→c) for all c∈K and apply it on (a→b)→a, we obtain that

(a→b)→a≼(a→b)→((a→b)→b)

so that

ϵ((a→b)→((a→b)→b))≥ϵ((a→b)→u).

Substituting a→b in place of a in Theorem 4.3 gives

$$\begin{array}{c} \epsilon \left(\left(a\to b\right)\to b\right)\geq \epsilon \left(\left(a\to b\right)\to \left(\left(a\to b\right)\to b\right)\right)\\ \;\geq \epsilon \left(\left(a\to b\right)\to a\right). \end{array}$$

Applying the condition of Definition 3.5 we get

ϵ(b)≥ϵ(a→b)∧ϵ((a→b)→b)≥ϵ(a→b)∧ϵ((a→b)→a).

Also, from the fact that b·a≼b we get b≼a→b. Thus, ϵ(a→b)≥ ϵ(b). Following this we have ϵ(b)≥ϵ((a→b)→a) . This proves the theorem.      □

Theorem 4.19. *A comparative fuzzy filter of a QORS*, K *is an implica- tive fuzzy filter.*

Proof.
Suppose *ϵ* be a comparative fuzzy filter of K. Since

ϵ(a→(b→c))=ϵ(b→(a→c))foranya,b,c∈K,

we have

ϵ(a→b)∧ϵ(a→(b→c))≤ϵ(a→(b→c))=ϵ(b→(a→c)).

From the fact that *b · a* ≼ *b* implies *b* ≼ *a → b* and ( *a → c*) *.a* ≼ *a → c* implies *a → c* ≼≼ *a →* ( *a → c*) we obtain that

b→(a→c)≼(a→b)→(a→(a→c)).

So, considering the condition *a → b* = (( *a → b*) *→ b*) *→ b* and applying Theorem 4.13 we obtain the following:

$$\begin{array}{ll} \epsilon \left(b\to \left(a\to c\right)\right) & \leq \epsilon \left(\left(a\to b\right)\to \left(a\to \left(a\to c\right)\right)\right)\\ & \leq \epsilon \left(a\to \left(a\to c\right)\right)\\ \; & =\epsilon \left(a\to \left(\left(\left(a\to c\right)\to c\right)\to c\right)\right)\\ \; & =\epsilon \left((\left(a\to c\right)\to c)\right)\to \left(a\to c\right))\\ \; & \leq \epsilon \left(a\to c\right). \end{array}$$

Thus,

ϵ(a→c)≥ϵ(a→b)∧ϵ(a→(b→c)).

Therefore, *ϵ* is an implicative fuzzy filter of K .      □

Theorem 4.20. *The family* $F_{c}\left(K\right)$ *of all comparative fuzzy filters in a QORS*, K *forms a complete lattice.*

Proof.
The proof follows from Theorem 3.7 and Theorem 4.15.      □

Corollary 4.21. *Let* K *be a QORS. For any fuzzy subset ϵ of* K *, there is a unique minimum comparative fuzzy filter in* K *that contains* ϵ *.*

Theorem 4.22. *A fuzzy subset* ϵ *of a QORS*, K *is a comparative fuzzy filter iff* $\epsilon_{\alpha }$ *for all* α∈[0,1], *is a comparative filter of* K *.*

Proof.
Suppose ϵ is a comparative fuzzy filter of K . Let $a\to \left(\left(b\to c\right)\to b\right)\in \epsilon_{\alpha }$ and $a\in \epsilon_{\alpha }\;$ for all a,b,c∈K .

Since

ϵ(b)≥ϵ(a)∧ϵ(a→((b→c)→b))≥α,

we have $b\in \epsilon_{\alpha }$ . So, $\epsilon_{\alpha }$ is a comparative filter of K . Conversely, suppose $\mu_{\alpha }$ is a comparative filter of K . Let

ϵ(a→((b→c)→b))∧ϵ(a)=α.

This implies that

ϵ(a→((b→c)→b))≥αandϵ(a)≥α

so that

$$a\to \left(\left(b\to c\right)\to b\right)\in \epsilon_{\alpha }\;\text{and}\;a\in \epsilon_{\alpha }.$$

Since $\epsilon_{\alpha }$ is a comparative filter, $b\in \epsilon_{\alpha }$ i.e.,

ϵ(b)≥α=ϵ(a)∧ϵ(a→((b→c)→b)).

Therefore, ϵ is a comparative fuzzy filter of K .      □

Definition 4.23. A fuzzy filter ϵ of a QORS, K satisfying the condition ϵ((a→b)→b)≥ϵ(c)∧ϵ(c→((b→a)→a)) for all a,b,c∈K , is said to be a normal fuzzy filter of K .

A QORS, K satisfying the condition (a→b)→b≡≼(b→a)→a for all a,b∈K , is said to be strong QORS. ^14^

Theorem 4.24. *Let* K *be a strong QORS and* ϵ *be a fuzzy filter of* K *. Then* ϵ *is a normal fuzzy filter of* K *.*

Proof.
Suppose K be a strong QORS and ϵ be a fuzzy filter of K . Thus, from the condition of Definition 3.5 and for a,b,c∈K as (a→b)→b=(b→a)→a , we have

$$\begin{array}{c} \epsilon \left(\left(a\to b\right)\to b\right)\;\geq \epsilon \left(c\right)\wedge \epsilon \left(c\to \left(\left(a\to b\right)\to b\right)\right)\\ \;=\epsilon \left(c\right)\wedge \epsilon \left(c\to \left(\left(b\to a\right)\to a\right)\right). \end{array}$$

Therefore, ϵ is a normal fuzzy filter of K .      □

Theorem 4.25. *A fuzzy filter* ϵ *of a QORS*, K *is a normal fuzzy filter of* K *if and only if* ϵ((a→b)→b)≥ϵ((b→a)→a).

Proof.
Suppose ϵ be a normal fuzzy filter of K . From Lemma 4.4,

ϵ((a→b)→b)≥ϵ(1→((b→a)→a))=ϵ((b→a)→a)), for all a,b∈K.

Conversely, suppose that

ϵ((a→b)→b)≥ϵ((b→a)→a), for all a,b∈K. Since ϵ is a fuzzy filter of K from Definition 3.5, we have

ϵ((b→a)→a)≥ϵ(c)∧ϵ(c→((b→a)→a)).

Thus,

ϵ((a→b)→b)≥ϵ(c)∧ϵ(c→((b→a)→a)).

So, ϵ is a normal fuzzy filter of K .      □

Theorem 4.26. *A comparative fuzzy filter of a QORS*, K *is a normal fuzzy filter of* K *.*

Proof.
Suppose ϵ be a comparative fuzzy filter of K . For any a,b,c∈K , we have a·(b→a)≼a so that a≼(b→a)→a. Thus,

$$\begin{array}{c} \left(\left(b\to a\right)\to a\right)\to b≼a\to b.\\ \Rightarrow \left(a\to b\right)\to b≼\left(\left(\left(b\to a\right)\to a\right)\to b\right)\to b. \end{array}$$

Also, as b≼(b→a)→a, we have

(((b→a)→a)→b)→b≼(((b→a)→a)→b)→((b→a)→a),

which shows that

(a→b)→b≼(((b→a)→a)→b)→((b→a)→a).

Since ϵ is comparative fuzzy filter,

$$\begin{array}{c} \epsilon \left(\left(b\to a\right)\to a\right)\geq \epsilon \left(\left(\left(\left(b\to a\right)\to a\right)\to b\right)\to \left(\left(b\to a\right)\to a\right)\right)\\ \;\geq \epsilon \left(\left(a\to b\right)\to b\right). \end{array}$$

So, as ϵ is a fuzzy filter we have

ϵ((a→b)→b)≥ϵ(c)∧ϵ(c→((a→b)→b)).

Thus,

ϵ((b→a)→a)≥ϵ(c)∧ϵ(c→((a→b)→b)).

Hence, ϵ is normal fuzzy filter of K .      □

Theorem 4.27. *A normal fuzzy filter of a QORS*, K *is a comparative fuzzy filter if it is an implicative fuzzy filter of* K *.*

Proof.
Suppose ϵ be both a normal fuzzy filter and an implicative fuzzy filter of a QORS K . Clearly, for any a,b∈K ; if a≼b , then ϵ(a)≤ϵ(b) . Since ϵ is a fuzzy filter of K , to show that *ϵ* is a comparative fuzzy filter of K by Theorem 4.16 it suffices to show that ϵ(b)≥ϵ((b→c)→b) for all b,c∈K . From the condition of Definition 3.5, we have

ϵ(b)≥ϵ(a→b)∧ϵ((a→b)→b).

Since a≼b→a and hence (b→a)→b≼a→b, we get

ϵ(a→b)≥ϵ((b→a)→b).

Thus,

ϵ(b)≥ϵ((b→a)→b)∧ϵ((a→b)→b).

And using Theorem 4.3 and Theorem 4.25, we have

$$\begin{array}{c} \epsilon \left(b\right)\geq \epsilon \left(\left(b\to a\right)\to b\right)\wedge \epsilon \left(\left(b\to a\right)\to a\right)\\ \;\geq \epsilon \left(\left(b\to a\right)\to b\right)\wedge \epsilon \left(\left(b\to a\right)\to \left(\left(b\to a\right)\to a\right)\right). \end{array}$$

Now, b≼(b→a)→a which implies that

(b→a)→b≼(b→a)→((b→a)→a).

So,

ϵ(b)≥ϵ((b→a)→b)∧ϵ((b→a)→b)=ϵ((b→a)→a).

Therefore, ϵ is a comparative fuzzy filter of K .      □

## 5. Fantastic and Associative Fuzzy Filters of Pseudo Quasi-Ordered Residuated System

This section examines the notions of fantastic and associative fuzzy filters within a pseudo quasi-ordered residuated framework. Additionally, their relationships with implicative and comparative fuzzy filters are explored.

Definition 5.1. A fuzzy subset ϵ of a Psedo Quasi-Ordered Residuated System(PsQORS) K = ( *K,., →,* ⇝ *,* 1 *,* ≼ ) is a fuzzy filter of K if it satisfies the following conditions:

(1) If a≼b, then ϵ(b)≥ϵ(a) ,
(2)
ϵ(b)≥ϵ(a)∧ϵ(a→b),

(3)
ϵ(b)≥ϵ(a)∧ϵ(a⇝b),

for any a,b∈K.

We refer the operations, ‘ · ’ as (non-commutative) multiplication, to ‘ *→*’ as its left residuum and ‘⇝’ to as right residuum.

For any a,b∈K , let a≼b implies that ϵ(b)≥ϵ(a). Since a≼a→b, we have ϵ(a→b)≥ϵ(a). Thus ϵ(a→b)∧ϵ(a)=ϵ(a) so that ϵ(b)≥ϵ(a)∧ϵ(a→b) , which shows that (1) implies (2) in Definition 4.29.

Lemma 5.2. *Let* ϵ *be a fuzzy subset of a PsQORS*, K *satisfying (QF). Then, the following conditions hold:*

(1)
ϵ(1→a)=ϵ(a),

(2)
ϵ(1⇝a)=ϵ(a) *for all* a∈K *.*

Theorem 5.3. *Suppose* ϵ *be a fuzzy set of a PsQORS*, K *with* a,b∈K *such that* a≼b *implies that* ϵ(a)≤ϵ(b) *. Then the following conditions hold:*

(1)
ϵ(a→(1→b))=ϵ(a→b)

(2)
ϵ(a⇝(1⇝b))=ϵ(a⇝b).

Definition 5.4. A fuzzy subset ϵ of a PsQORS, K is said to be an implicative fuzzy filter of K if it satisfies the following conditions:

(1) If a≼b, then ϵ(b)≥ϵ(a),

(2)
ϵ(b)≥ϵ(a)∧ϵ(a→((b→c)⇝b)),

(3)
ϵ(b)≥ϵ(a)∧ϵ(a⇝((b⇝c)→b))

for any a,b,c∈K .

Theorem 5.5. *Let* ϵ *be a fuzzy subset of a PsQORS*, K *. Then* ϵ *is an implicative fuzzy filter of* K *if and only if for all* a,b∈K, *the following conditions hold:*

(1)
ϵ(a)≥ϵ((a→b)⇝a),

(2)
ϵ(a)≥ϵ((a⇝b)→a).

Proof.
Suppose ϵ be an implicative fuzzy filter of a PsQORS, K for all a,b,c∈K . Then,

ϵ(a)≥ϵ(1)∧ϵ(1→((a→b)⇝a))

and from Lemma 4.4, we get ϵ((a→b)⇝a)=ϵ(1→((a→b)⇝a)) . Thus, ϵ(a)≥ϵ((a→b)⇝a). Applying the same procedure gives condition (2).

Conversely, suppose conditions (1) and (2) are satisfied. Definition 4.29 (2) asserts that

ϵ(a)≥ϵ((a→b)⇝a)≥ϵ(c)∧ϵ(c→((a→b)⇝a)).

Similarly, from (3) of Definition 4.29 we get

ϵ(a)≥ϵ((a⇝b)→a)≥ϵ(c)∧ϵ(c⇝((a⇝b)→a)).

Hence, ϵ is an implicative fuzzy filter of K .      □

Theorem 5.6. *Every implicative fuzzy filter of a PsQORS*, K *is a fuzzy filter of* K *.*

Proof.
Suppose ϵ be an implicative fuzzy filter of K and a,b∈K . Then, from (2) of Definition 4.31 we get

ϵ(b)≥ϵ(a)∧ϵ(a→((b→1)⇝b))≥ϵ(a)∧ϵ(a→b).

Similarly, from (3) of Definition 4.31 we get

ϵ(b)≥ϵ(a)∧ϵ(a⇝((b⇝1)→b))≥ϵ(a)∧ϵ(a⇝b).

Therefore, ϵ is a fuzzy filter of K .      □

Definition 5.7. A fuzzy subset ϵ of a PsQORS, K is said to be a comparative fuzzy filter of K if it satisfies the following conditions:

(1) If a≼b, then ϵ(b)≥ϵ(a) ,
(2)
ϵ(a→c)≥ϵ(a⇝b)∧ϵ(a→(b→c)),

(3)
ϵ(a⇝c)≥ϵ(a→b)∧ϵ(a⇝(b⇝c))

for any a,b,c∈K.

Theorem 5.8. *A comparative fuzzy filter of a PsQORS*, K *is a fuzzy filter of* K *.*

Proof.
Let ϵ be a comparative fuzzy filter of K and a,b,c∈K . Applying Lemma 4.28 and Definition 4.34 (2) and (3) together shows that

$$\begin{array}{l} \epsilon \left(c\right)\geq \epsilon \left(1\to c\right)\\ \;\geq \epsilon \left(1⇝b\right)\wedge \epsilon \left(1\to \left(b\to c\right)\right)\\ \;\geq \epsilon \left(b\right)\wedge \epsilon \left(b\to c\right), \end{array}$$

and

$$\begin{array}{l} \epsilon \left(c\right)\geq \epsilon \left(1⇝c\right)\\ \;\geq \epsilon \left(1\to b\right)\wedge \epsilon \left(1⇝\left(b⇝c\right)\right)\\ \;\geq \epsilon \left(b\right)\wedge \epsilon \left(b⇝c\right). \end{array}$$

Hence, ϵ is a fuzzy filter of K .      □

Definition 5.9. A fuzzy subset ϵ of a PsQORS, K is said to be a fantastic fuzzy filter of K if it satisfies the following conditions:

(1) If a≼b , then ϵ(b)≥ϵ(a),

(2)
ϵ(((b→a)⇝a)→b)≥ϵ(c)∧ϵ(c→(a→b)),

(3)
ϵ(((b⇝a)→a)⇝b)≥ϵ(c)∧μ(c⇝(a⇝b))

for any a,b,c∈K.

Theorem 5.10. *A fantastic fuzzy filter of a PsQORS*, K *is a fuzzy filter of* K *.*

Proof.
Suppose ϵ be a fantastic fuzzy filter of a PsQORS, K . From Definition 4.36 (2), for any b,c∈K we have

ϵ(b)≥ϵ(((b→1)⇝1)→b)≥ϵ(c)∧ϵ(c→b).

Similarly, from Definition 4.36 (3) we get

ϵ(b)≥ϵ(((b⇝1)→1)⇝b)≥ϵ(c)∧ϵ(c⇝b).

Therefore, ϵ is a fuzzy filter of K .      □

Theorem 5.11. *A fuzzy filter of a PsQORS*, K *is a fantastic fuzzy filter of K if and only if the conditions:*

(1)
ϵ(((b→a)⇝a)→b)≥ϵ(b→a) *and*
(2)
ϵ(((b⇝a)→a)⇝b)≥ϵ(b⇝a)

*are satisfied for all* a,b∈K *.*

Proof.
Suppose ϵ be a fantastic fuzzy filter of a PsQORS, K . Clearly, from Lemma 4.28, ϵ(1→(a→b))=ϵ(a→b) for all a,b∈K . By Definition 4.34 (3), we have

ϵ(((b⇝a)→a)⇝b)≥ϵ(1)∧ϵ(1⇝(a⇝b))=ϵ(b⇝a).

Similarly, ϵ(1⇝(a⇝b))=ϵ(a⇝b) for all a,b∈K . So,

ϵ(((b→a)⇝a)→b)≥ϵ(1)∧ϵ(1→(a→b))=ϵ(b→a).

Conversely, suppose *ϵ* be a fuzzy filter satisfying conditions (1) and (2). Then, by (2) of Definition 4.29, we have

ϵ(a→b)≥ϵ(c)∧ϵ(c→(a→b))

and by (1) we get

ϵ(((b→a)⇝a)→b)≥ϵ(a→b)≥ϵ(c∧(c→(a→b))).

Also, from (3) of Definition 4.29, we have

ϵ(a⇝b)≥ϵ(c∧(c→(a⇝b)))

and by (2) we get

ϵ(((b⇝a)→a)⇝b)≥ϵ(a⇝b)≥ϵ(c∧(c⇝(a⇝b))).

Therefore, ϵ is a fantastic fuzzy filter of K .      □

Definition 5.12. A fuzzy subset ϵ of a PsQORS, K is said to be an associative fuzzy filter of K if it satisfies the following conditions:

(1) If a≼b, then ϵ(b)≥ϵ(a) ,
(2)
ϵ(c)≥ϵ(a⇝b)∧ϵ(a→(b→c)) ,
(3)
ϵ(c)≥ϵ(a→b)∧ϵ(a⇝(b⇝c))

for any a,b,c∈K.

Theorem 5.13. *An associative fuzzy filter of a PsQORS*, K *is a fuzzy filter of* K *.*

Proof.
Let ϵ be an associative fuzzy filter of K and a,b,c∈K . From Lemma 4.28, we have

$$\begin{array}{l} \epsilon \left(c\right)\geq \epsilon \left(1⇝b\right)\wedge \epsilon \left(1\to \left(b\to c\right)\right)\\ \;\geq \epsilon \left(b\right)\wedge \epsilon \left(b\to c\right), \end{array}$$

and

$$\begin{array}{l} \epsilon \left(c\right)\geq \epsilon \left(1\to b\right)\wedge \epsilon \left(1⇝\left(b⇝c\right)\right)\\ \;\geq \epsilon \left(b\right)\wedge \epsilon \left(b⇝c\right). \end{array}$$

Hence, ϵ is a fuzzy filter of K .      □

Theorem 5.14. *An associative fuzzy filter* ϵ *of a PsQORS*, K *is an implicative fuzzy filter of* K *.*

Proof.
Suppose ϵ be an associative fuzzy filter of K . By Theorem 4.30, for any a,b∈K , we have

ϵ((a→b)⇝(ϵ⇝a))≥ϵ((a→b)⇝a) .

Applying (3) of Definition 4.39 gives

$$\begin{array}{l} \epsilon \left(a\right)\geq \epsilon \left(\left(a\to b\right)⇝\left(\epsilon ⇝a\right)\right)\wedge \epsilon \left(\left(a\to b\right)\to 1\right)\\ \;=\epsilon \left(\left(a\to b\right)⇝\left(1⇝a\right)\right)\wedge \epsilon \left(1\right)\\ \begin{array}{c} \;=\epsilon \left(\left(a\to b\right)⇝\left(1⇝a\right)\right)\\ \;=\epsilon \left(\left(a\to b\right)⇝a\right). \end{array} \end{array}$$

Also, ϵ((a⇝b)→(1→a))≥ϵ((a⇝b)→a). So, applying (2) of Definition 4.39 gives

$$\begin{array}{l} \epsilon \left(a\right)\geq \epsilon \left(\left(a⇝b\right)\to \left(1\to a\right)\right)\wedge \mu \left(\left(a⇝b\right)⇝1\right)\\ \;=\epsilon \left(\left(a⇝b\right)\to \left(1\to a\right)\right)\wedge \epsilon \left(1\right)\\ \begin{array}{c} \;=\epsilon \left(\left(a⇝b\right)\to \left(1\to a\right)\right)\\ \;=\epsilon \left(\left(a⇝b\right)\to a\right). \end{array} \end{array}$$

Hence, by Theorem 4.32 ϵ is an implicative fuzzy filter of K .      □

Theorem 5.15. *An associative fuzzy filter* ϵ *of a PsQORS*, K *is a comparative fuzzy filter of* K *.*

Proof.
Suppose ϵ be an associative fuzzy filter of K . Let a,b,c∈K. Clearly, c≼a→c so that

ϵ(a→c)≥ϵ(c)≥ϵ(a⇝b)∧ϵ(a→(b→c)),

which shows that (2) of Definition 4.34 is valid. Similarly, c≼a⇝c

so that

ϵ(a⇝c)≥ϵ(c)≥ϵ(a→b)∧ϵ(a⇝(b⇝c)),

which shows that (3) of Definition 4.34 is valid. Thus, ϵ is a comparative fuzzy filter of K. □

## 6. Conclusions

This paper introduces the notions of implicative fuzzy filters within a quasi-ordered (pseudo quasi-ordered) residuated framework, K . It establishes sufficient conditions for a fuzzy filter in a QORS(PsQORS) K to qualify as an implicative fuzzy filter. Additionally, the concept of comparative fuzzy filters in QORS(PsQORS) is examined, along with the necessary and sufficient criteria for a fuzzy filter to attain this classification. Furthermore, the study demonstrates that the collection of all comparative fuzzy filters in a quasi-ordered residuated system K forms a complete lattice, and the interplay between implicative and comparative fuzzy filters is analyzed. The paper also investigates fantastic and associative fuzzy filters within a pseudo quasi-ordered residuated structure. These results can be extended to the concept of intuitionistic and neutrosophic fuzzy logics, fuzzy soft sets, anti-fuzzy sets and other forms of filters in QORS(PsQORS).

## Data Availability

No data are associated with this manuscript.

## References

[1] Bonzio S Chajda I : Residuated relational systems. *Asian-European J. Math.* 2018;11(2):1850024. 10.1142/S1793557118500249

[2] Romano DA : Filters in residuated relational system ordered under quasi-order. *Bull. Int. Math. Virtual Inst.* 2020;10(3):529–534.

[3] Romano DA : Ideals in quasi-ordered residuated system. *Contrib. Math.* 2021;3:68–76.

[4] Romano DA : pseudo quasi-ordered residuated system. *Pan-Am. J. Math.* 2022;1(1):1–12. 10.28919/cpr-pajm/1-12

[5] Romano DA : Implicative filters in quasi-ordered residuated system. *Proyecciones J. Math.* (To appear).

[6] Romano DA : Associated filters in quasi-ordered residuated systems. *Contrib. Math.* 2020;1:22–26.

[7] Romano DA : Comparative filters in quasi-ordered residuated system. *Bull. Int. Math. Virtual Inst.* 2021;11(1):177–184. 10.7251/BIMVI2101177R

[8] Zadeh LA : Fuzzy sets. *Inf. Control.* 1965;8:338–353. 10.1016/S0019-9958(65)90241-X

[9] Rosenfeld A : Fuzzy groups. *J. Math. Anal. Appl.* 1971;35:512–517. 10.1016/0022-247X(71)90199-5

[10] Yuan B Wu W : Fuzzy ideals on a distributive lattice. *Fuzzy Sets Syst.* 1990;35:231–240. 10.1016/0165-0114(90)90196-D

[11] Swamy UM Raju DV : Fuzzy ideals and congruences of lattices. *Fuzzy Sets Syst.* 1998;95:249–253. 10.1016/S0165-0114(96)00310-7

[12] Sundar Raj CS Amare NT Swamy UM : Fuzzy prime ideals of ADL’s. * Int. J. Comput. Sci. Appl. Math.* 2018;4:32–36.

[13] Swamy UM Santhi Sundar Raj C Teshale AN : L-Fuzzy Filters of Almost Distributive Lattices. *IJMSC.* 2018;8(1):35–43. 10.26708/IJMSC.2017.1.8.05

[14] Romano DA : strong quasi-ordered residuated system. *Open J. Math. Sci.* 2021;5:73–79. 10.30538/oms2021.0146

