# Supplementary material for: The Notions of Fuzzy Set on Pseudo Quasi Ordered Residuated Systems
Source: F1000Res. 2025 Jul 24;14:566. Originally published 2025 Jun 6. [Version 2] doi: 10.12688/f1000research.165259.2 (PMC12247480; doi:10.12688/f1000research.165259.2)
Supplement: Peer review history

# Reviewer report: Reviewer response for version 1

**DOI:** 10.5256/f1000research.181869.r390734

**Published:** 2025-07-21

**Authors:** Gerima Tefera

**Affiliations:** 1 Wollo University, Dessi, Ethiopia

1. Abstract Grammar and Punctuation: Corrected article usage ("the concept" → "the concepts"),removed unnecessary spaces (e.g., "K ."), and ensured proper use of commas and conjunctions. Flow of Ideas: Improved clarity and sentence transitions to make the logical progressions moother. Originality: The content appears original and academic, suitable for a research context. There are no signs of plagiarism in the phrasing or structure.

 2. Introduction A. Orginality The content appears original and aligned with standard mathematical discourse in fuzzyalgebra. The concepts (like comparative, implicative, and associative fuzzy filters) are commonin fuzzy order theory, so their presence alone does not suggest plagiarism.Textual Originality:2The wording does not match any commonly known or published paper verbatim. It appears to be original or paraphrased appropriately if based on prior word.Avoid repeating long phrases like “quasi-ordered residuated system” unnecessarily. “the sufficient conditions” → should be “sufficient conditions” .“...comparative, normal, implicative and associative fuzzy filters within a quasi-ordered residuated system.”Repeating “within a quasi-ordered residuated system” twice in consecutive sentences isredundant. So adjust this conditions. “vice-versa” Although acceptable, the preferred form in academic writing is “vice versa”(without hyphen).Missing serial comma in lists (optional in British English, recommended in American English forclarity): “comparative, normal, implicative, and associative fuzzy filters”-All references are cited except 7 and 14 .So include these incitation.

 3. Preliminaries This section is well stated and cited.4. Main ResultInsection 3 line 1for all a , ,b ∈ K the condition u ≼ v ∈ ∈ can bewritten ∈ ∈ ∈ is denoted by (QF).And it has to be stated either as definition or as Lemma because this idea usedthroughout the paper3-Give captionfor each table.-In example 4.2 lne 4 is not clear justify it.In proof of Theorem 3.10 line 1 there is a missing term and adjust it.5. ConclusionIt is well written and cites future work also.General CmmentThe research is the original work of authors and it has new results.I recommend for the publication after incorporating the comments given above.

Is the work clearly and accurately presented and does it cite the current literature?

Yes

If applicable, is the statistical analysis and its interpretation appropriate?

Not applicable

Are all the source data underlying the results available to ensure full reproducibility?

Partly

Is the study design appropriate and is the work technically sound?

Yes

Are the conclusions drawn adequately supported by the results?

Yes

Are sufficient details of methods and analysis provided to allow replication by others?

No

Reviewer Expertise:

On Fuzzy algebra and related

I confirm that I have read this submission and believe that I have an appropriate level of expertise to confirm that it is of an acceptable scientific standard.

# Reviewer report: Reviewer response for version 1

**DOI:** 10.5256/f1000research.181869.r390737

**Published:** 2025-07-09

**Authors:** Wondwosen Zemene Norahun

**Affiliations:** 1 University of Gondar, Gondar, Ethiopia

In this work, the authors have studied the concept of fuzzy filters in a quasi-ordered residuated system. They also investigate various types of fuzzy filters such as 2-fuzzy filters, normal fuzzy filters, implicative fuzzy filters, and associative fuzzy filters. Furthermore, they examine their interrelationships and prove that the class of all fuzzy filters of a pseudo quasi-ordered residuated system forms a complete lattice. All results presented in this paper are mathematically correct and clearly written. However, there are some typographical errors: for example, in the title, “notions” should be corrected to “notion of...”. In general, I recommend this paper for indexing.

Is the work clearly and accurately presented and does it cite the current literature?

Yes

If applicable, is the statistical analysis and its interpretation appropriate?

Not applicable

Are all the source data underlying the results available to ensure full reproducibility?

Yes

Is the study design appropriate and is the work technically sound?

Yes

Are the conclusions drawn adequately supported by the results?

Yes

Are sufficient details of methods and analysis provided to allow replication by others?

Yes

Reviewer Expertise:

Fuzzy theory, Lattice theory and Universal algebra.

I confirm that I have read this submission and believe that I have an appropriate level of expertise to confirm that it is of an acceptable scientific standard.

# Reviewer report: Reviewer response for version 1

**DOI:** 10.5256/f1000research.181869.r390735

**Published:** 2025-07-09

**Authors:** Hemavathi P, Dr. Vinod Kumar R

**Affiliations:** 1 Saveetha Institute of Medical and Technical Sciences (SIMATS), Thandalam, India; 2 Mathematics, Rajalakshmi Engineering College, Thandalam/Chennai, Tamil Nadu, India

This manuscript introduces and investigates a variety of fuzzy filters such as implicative, comparative, normal, fantastic, and associative fuzzy filters within the framework of quasi-ordered and pseudo quasi-ordered residuated systems (QORS and PsQORS). The paper makes a substantial contribution to the ongoing theoretical development of fuzzy algebraic systems and residuated logic structures.

**Strengths**

- The paper presents **original and rigorous definitions** of new fuzzy filter types and establishes their interrelationships through a series of well-formulated theorems.
- The **proofs are generally sound**, logically structured, and supported by appropriate examples.
- The inclusion of results on **complete lattice structures** of fuzzy filters adds further value and shows depth in mathematical abstraction.
- The extension from QORS to **pseudo quasi-ordered systems (PsQORS)** is meaningful and handled with consistency.

**Concerns and Suggestions**

- The manuscript contains several **grammatical inconsistencies and awkward phrasings** (e.g., “psedoQuasi-Ordered” instead of “pseudo quasi-ordered”), which may impede readability for a broader audience. A thorough language edit is recommended.
- Some symbols (e.g., "μ", various implications like “!”, “ *→* ”) are introduced without clear distinction or may be misinterpreted by readers unfamiliar with the notational conventions. Providing a **notation table** or clarifying these in the preliminaries would help.
- While the theoretical contribution is strong, the manuscript would benefit from a **discussion on potential applications**, such as implications in fuzzy logic systems, decision-making, or soft computing frameworks.

**Minor Points**

- Typos and formatting errors are scattered throughout the manuscript and should be carefully corrected (e.g., spacing in equations, punctuation in theorem statements).
- Consistency in theorem numbering and reference citations would improve professional presentation.

**Recommendation:**

**Minor Revision**

 The article is mathematically rich and presents meaningful theoretical advancements. I recommend minor revisions primarily to improve language, clarity, and presentation. With these improvements, the paper would be suitable for indexing.

Is the work clearly and accurately presented and does it cite the current literature?

Yes

If applicable, is the statistical analysis and its interpretation appropriate?

Not applicable

Are all the source data underlying the results available to ensure full reproducibility?

No source data required

Is the study design appropriate and is the work technically sound?

Yes

Are the conclusions drawn adequately supported by the results?

Yes

Are sufficient details of methods and analysis provided to allow replication by others?

Yes

Reviewer Expertise:

Fuzzy algebra, Neutrosophic sets, Fuzzy Decision Making

We confirm that we have read this submission and believe that we have an appropriate level of expertise to confirm that it is of an acceptable scientific standard.
